# The Healthy Steps Study: A randomized controlled trial of a pedometer-based Green Prescription for older adults. Trial protocol

**DOI:** 10.1186/1471-2458-9-404

**Published:** 2009-11-01

**Authors:** Gregory S Kolt, Grant M Schofield, Ngaire Kerse, Nicholas Garrett, Philip J Schluter, Toni Ashton, Asmita Patel

**Affiliations:** 1 School of Biomedical and Health Sciences, University of Western Sydney, Sydney, Australia; 2 Centre for Physical Activity and Nutrition Research, Auckland University of Technology, Auckland, New Zealand; 3 Department of General Practice and Primary Health Care, School of Population Health, University of Auckland, Auckland, New Zealand; 4 School of Public Health and Psychosocial Studies, Auckland University of Technology, Auckland, New Zealand; 5 The University of Queensland, School of Nursing and Midwifery, Australia; 6 Health Systems Section, School of Population Health, University of Auckland, Auckland, New Zealand

## Abstract

**Background:**

Graded health benefits of physical activity have been demonstrated for the reduction of coronary heart disease, some cancers, and type-2 diabetes, and for injury reduction and improvements in mental health. Older adults are particularly at risk of physical inactivity, and would greatly benefit from successful targeted physical activity interventions.

**Methods/Design:**

The Healthy Steps study is a 12-month randomized controlled trial comparing the efficacy of a pedometer-based Green Prescription with the conventional time-based Green Prescription in increasing and maintaining physical activity levels in low-active adults over 65 years of age. The Green Prescription interventions involve a primary care physical activity prescription with 3 follow-up telephone counselling sessions delivered by trained physical activity counsellors over 3 months. Those in the pedometer group received a pedometer and counselling based around increasing steps that can be monitored on the pedometer, while those in the standard Green Prescription group received counselling using time-based goals. Baseline, 3 month (end of intervention), and 12 month measures were assessed in face-to-face home visits with outcomes measures being physical activity (Auckland Heart Study Physical Activity Questionnaire), quality of life (SF-36 and EQ-5D), depressive symptoms (Geriatric Depression Scale), blood pressure, weight status, functional status (gait speed, chair stands, and tandem balance test) and falls and adverse events (self-report). Utilisation of health services was assessed for the economic evaluation carried out alongside this trial. As well, a process evaluation of the interventions and an examination of barriers and motives for physical activity in the sample were conducted. The perceptions of primary care physicians in relation to delivering physical activity counselling were also assessed.

**Discussion:**

The findings from the Healthy Steps trial are due in late 2009. If successful in improving physical activity in older adults, the pedometer-based Green Prescription could assist in reducing utilisation of health services and improve cardiovascular health and reduction of risk for a range of non-communicable lifestyles diseases.

**Trial registration:**

Australian and New Zealand Clinical Trials Registry ACTRN012606000023550

## Background

Many older adults are at risk of lifestyle-related diseases because of a lack of regular physical activity. Physical inactivity has both direct and indirect costs to the health of the population. Evidence shows the graded health benefits of physical activity for the reduction of coronary heart disease, some cancers, type-2 diabetes, obesity, osteoporosis, and injury (including falls in the elderly) [1]. Physical activity participation by older adults can also improve quality of life and physical and psychological function (including depression), facilitate independent living, and reduce the risk of dementia [2-5]. Improved physical and psychological functioning through regular physical activity also results in overall improved quality of life in older adults [6]. Older adults are particularly at risk of inactivity [7], and they also contribute disproportionately to the burden on health care systems [8].

The Green Prescription is a current successful strategy to deliver physical activity advice in New Zealand [9,10]. It is an activity-based prescription program administered through Primary Care settings with the support of trained telephone counselors. The Green Prescription is based around achieving daily time-based activity goals on the basis of the US Surgeon General's recommendation, and the national physical activity guidelines of 30 minutes of moderate activity on most, if not all, days of the week. The program is efficacious in increasing and maintaining physical activity 12 months post prescription [9] and is cost-effective both in terms of relative cost per quality adjusted life year [11] and per successful treatment [12].

Research has also shown that incidental physical activity may be an important contributor to achieving health benefits, especially for older adults [13]. Pedometer-based interventions have been suggested as an effective way to maintain levels of activity conducive to health benefits, especially in older adults (as evidenced in a systematic review) [14]. Further, using a pedometer to monitor the accumulation of daily physical activity may be a superior motivation and adherence tool to time-based goal setting.

In this study we aimed to compare the efficacy of a pedometer-based Green Prescription with the conventional time-based Green Prescription in increasing and maintaining physical activity levels in low-active older adults. As well, the trial sought to examine the effects of the interventions on health-related quality of life, functional status, blood pressure, weight status, and health care utilisation and costs. Incremental cost-effectiveness and cost-utility ratios will also be estimated.

## Methods/Design

### Design

A randomized controlled trial design was used to investigate the efficacy and cost-effectiveness of a pedometer-based Green Prescription in improving physical activity levels and health outcomes in low active older adults compared with the usual (time-based) Green Prescription. Ethical approval to conduct the study has been granted by the Health and Disability Ethics Committees, Northern Y Regional Ethics Committee (Reference Number NTY/05/11/086).

### Participants

Participants were community dwelling adults aged 65 years and older living on the North Shore in Auckland, New Zealand, who were identified through their usual primary care physician's computer databases. Participants were eligible for inclusion in the trial if they were low-active (i.e., had not engaged in at least 150 minutes of moderate physical activity accumulated over at least 5 days in the previous week). Other inclusion criteria were being able to freely give informed consent, being able to communicate in English, ability to walk, and having no health conditions that contraindicate physical activity participation as judged by their general practitioner (primary care physician). Exclusion criteria were visual impairment to a level where a pedometer screen could not be read.

### Recruitment (see Figure 1)

**Figure 1:**
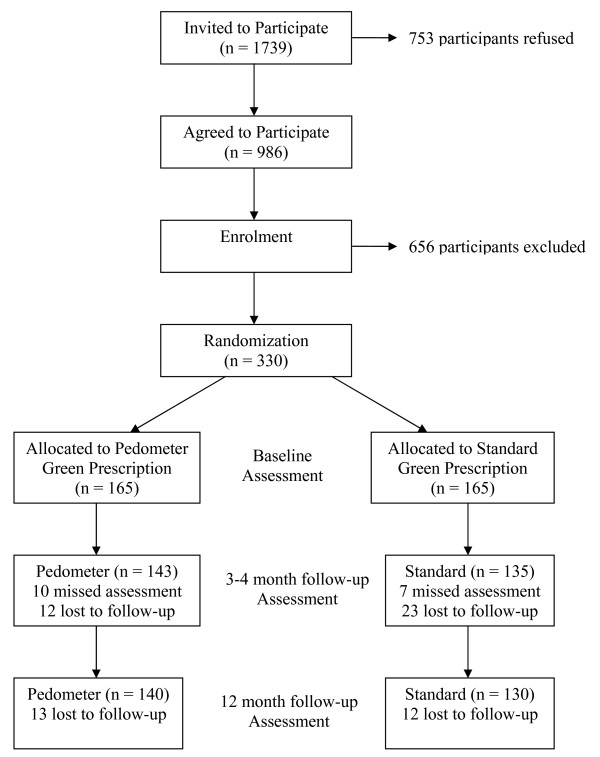
**Recruitment Flowchart and Identification of Eligible Population**.

Recruitment was modelled on that used in other primary care physical activity interventions for older adults [15,16]. A sample of 17 general practitioners from 10 medical practices on the North Shore of Auckland, New Zealand, accepted a faxed invitation to be involved in the study. These 10 practices (out of 38 that were initially invited) covered a range of socio-economic areas of this region of Auckland. The invitation was followed up with a telephone call to the general practitioners and a visit by one of the study staff. On agreement, a list of all patients 65 years and older was generated and the general practitioners and practice staff excluded patients who had a medical condition that would not allow safe participation in a physical activity program, dementia, and those who were not community-dwelling. An invitation letter, signed by the general practitioner, was mailed to patients with a reply-paid response card addressed to the research team. Those who returned their card indicating interest in the study were contacted by telephone to explain the study further and were screened for physical activity level with the question "As a rule, do you do at least half an hour of moderate or vigorous exercise (such as walking or a sport) on five or more days a week?" The positive predictive value of this question is 81% when identifying "less active" adults [9]. Health conditions contraindicated to physical activity were also screened using the Physical Activity Readiness Questionnaire (PAR-Q) [17]. This instrument is validated as an assessment tool for screening readiness for prescription of exercise, and is seen as appropriate for older adults. This tool was included to maximise participant safety. If the participant met the PAR-Q criteria, and was considered insufficiently active, the Research Officer arranged a home visit. If the patient had any contraindications to physical activity, general practitioner clearance was sought. Potential participants who were sufficiently active for health gain and/or who did not gain general practitioner clearance were excluded from participation in the trial. Patients who did not return their response card within three weeks were followed up with a single telephone call from the office of the general practitioner by the Research Officer to ascertain their willingness to participate in the study.

After making suitable arrangements, the Research Officer visited the patient in their home to answer any additional questions on the study, provide the information sheet, obtain informed consent, book a general practitioner appointment for commencement of the intervention, provide a falls and injuries calendar and reply-paid envelopes, and then take the following baseline measures: demographics questionnaire, blood pressure and current blood pressure medications (if applicable), lower extremity function measures, quality of life, physical activity, depressive symptoms, and self-reported health status.

During the general practitioner visit, the patient was randomized into either the standard Green Prescription or the pedometer Green Prescription group and commenced on the intervention through the initial prescription of physical activity. Those in the pedometer group were issued with a pedometer by the general practitioner or practice nurse and instructed in its use. At the end of the appointment the Green Prescription was faxed to the local Regional Sports Trust for continuation of the intervention. The Regional Sports Trust is as government-funded organization involved in provision of sports and physical activity opportunities and programs for the population of that region. General practitioners and other relevant practice staff participated in training relating to the administration of the intervention.

Recruitment was undertaken over 17 months from July 2006 to December 2007 to allow for the interventions to be delivered during all seasons. A total of 330 people were recruited into the trial. At 3 months, 278 remained in the study with 270 remaining at 12 months. Included in the 330 recruited participants were 31 couples (defined as living in the same household). In order to reduce the effects of contamination both individuals in the couple were allocated to the same intervention and were treated as clusters for the purposes of statistical analysis.

### Randomisation and Blinding

Randomisation was blocked and lists generated by an independent statistician (PJS) using specialist computer software. This list was used to allocate each participant to either the standard Green Prescription or pedometer Green Prescription group after enrolment in the study at the home visit. The allocation was advised to the general practitioner via a telephone call (from a Research Officer) to their reception staff before the participant attended their consultation for prescription of physical activity.

### Intervention Group

Participants in the pedometer Green Prescription group were provided with an initial prescription for physical activity by the general practitioner that was then followed up by 3 telephone counselling sessions by a physical activity counsellor at the Regional Sports Trust over a 3-month period (i.e., one call per month). The telephone counselling was based within a framework of the transtheoretical model of behaviour change [18] and used the principals of motivational interviewing [19] such as assisting patients to identify barriers and solutions to physical activity participation, and discussing positive aspects of participation in activity and negative aspects of not participating in enough activity. The strategies used were aimed at increasing participation in a variety of physical activities with an emphasis on walking. Goal setting was used as an important component of the intervention and participants were encouraged to increase their participation in activity by monitoring the number of steps recorded by their pedometer. In this way, the pedometer was used to provide immediate feedback to the participants regarding their accumulation of activity towards daily goals.

The content of each telephone counselling call was specific to each participant but typically included the following:

• Call 1 - Introductory call, information provision and goal setting (15-30 minutes).

• Call 2 - Follow-up call to assess progress (10-15 minutes).

• Call 3 - Final call to provide encouragement and relapse prevention (10-15 minutes).

### Control Group

Participants in the standard Green Prescription group participated in the same initial activity prescription by the general practitioner and the 3 telephone counselling sessions provided by the Regional Sports Trust with one difference: the physical activity prescription and counselling were based around accumulating activity in the context of time-related goals as opposed to pedometer step-related goals.

### Outcome Measures

Outcome measures were assessed at baseline, 3 months (end of intervention), and 12 months during home visits by the Research Officer at these times. All questionnaires were provided in English language only. The following were used.

#### Demographics

Demographics were collected at baseline and included self-identified ethnicity (allowing multiple ethnic groups), age, sex, marital status, education, occupation (lifetime and present), home ownership, and telephone and car ownership or access.

#### Physical activity

The Auckland Heart Study Physical Activity Questionnaire (AHSPAQ) was used to measure physical activity. The AHSPAQ is a validated [20] self-report questionnaire suitable for use with less active adults in primary care [21]. The AHSPAQ allows calculation of energy expenditure of leisure, occupational and domestic activity, rest, and sleep according to a standard physical activity compendium for energy expenditure values [22]. Software for the analysis of the AHSPAQ has been developed to calculate total and component energy expenditure, as well as duration-based physical activity outcomes [23].

Amount of walking on the previous day was assessed with a series of questions that requested participants to indicate the number of minutes they walked in 4 segments of the day: before breakfast, between breakfast and lunch, between lunch and dinner, and after dinner.

#### Quality of life

The SF-36 is a validated measure of health-related quality of life [24]. It assesses both mental health and physical health along eight dimensions; physical functioning, role physical, bodily pain, general health, vitality, social functioning, role emotional, and mental health. The SF-36 has been validated on New Zealand populations [25], has demonstrated suitability for use in elderly populations [26], and is associated with the stage of motivational readiness to changes in physical activity [27].

#### Functional status

Lower extremity function measures from the Short Physical Performance Battery (SPPB) (gait speed, chair stands, and tandem balance test) were used as a measure of functional capacity and disability risk [28-30].

#### Depressive symptoms

The Geriatric Depression Scale (GDS-15) is a well validated and extensively used scale [31] and was used to measure depressive symptoms in the Healthy Steps trial.

#### Blood pressure

Repeated blood pressures were measured and blood pressure medications were recorded.

#### Height and weight

Height was measured with a standard portable stadiometer, and weight was measured with digital scales. Body mass Index was calculated from height and weight.

#### Adverse events and associated costs

As a monitor of adverse events, all participants were monitored for one year using standardised reporting forms in the form of fridge diaries. These were 12-month diaries, (one page per month) that were filled in whenever a participant had a fall, injury, or musculoskeletal problem. All hospitalisations, bruises, skin lacerations, sutures, fractures, X-rays, and admissions as a result of these problems were also recorded on the falls form. As well, patient fees for general practitioner visits, consults with other health professionals, and costs associated with physical activity participation were collected on these forms for the economic evaluation. Forms were mailed to the research team monthly and a Research Officer telephoned the participant to follow-up on any injuries recorded and to prompt participants who had failed to send in their forms.

#### Self-reported health status

The EQ-5D is a generic measure of self-reported health status [32] and provides the information required to generate utility values for estimating quality-adjusted-life years (QALYs), the measure of outcome used in cost-utility analyses. The EQ-5D has been found to have acceptable reliability and validity in elderly patients [33,34].

#### Use of healthcare services

Summary information on the use of general practitioner services and inpatient hospital care, from general practitioner and New Zealand Health Information Service records, were obtained for the year prior to intervention and compared with the year following intervention delivery. Use of all other health services and any personal costs associated with the physical activity regime were collected on the monthly diaries described above.

#### Pedometer usage

At the end of the intervention (3 months) and at the 12 month follow-up a series of questions regarding pedometer usage were asked of all participants [35]. Questions related to knowledge of pedometers, wearing patterns over the previous period, and whether participants felt that the pedometer helped them increase their level of physical activity.

### Sample Size

Based on means, standard deviations, and change from previous trials, a sample of 137 per group would have at least 80% power at the 0.05 level of significance to detect a true difference in the change between the two groups of 5 points on the general health domain score of the SF-36, 1 hour of moderate physical activity, and a detectable difference of 15% of people meeting physical activity guidelines. To allow for an attrition rate of approximately 20% over 12 months, 165 participants were required in each group.

### Analysis

The primary analysis for this trial will be based on group allocation and the intention to treat principle. Apposite longitudinal methods, such as mixed-effects multilevel models and generalized estimating equation analysis will be used to compare the two treatment groups, accommodating the correlation between repeated observations and the natural clusterings and hierarchies in the sample [36]. Missing data will be assessed and multiple imputation undertaken, where necessary [37]. Model assumptions and diagnostics will be rigorously assessed [38].

An economic evaluation will form part of this trial. Incremental cost-effectiveness ratios will be calculated for selected outcome measures, such as cost per total energy expenditure achieved and cost per kilogram of weight lost, if any incremental change in outcome is statistically significant. For the cost-utility analyses, utility values will be calculated using the visual analogue scale of the EQ-5D. If the incremental gain in utility values is statistically significant, these utility values will be transformed into Quality Adjusted Life Years (QALY's) and the incremental cost per QALY will be calculated.

### Adjunct Studies

The Healthy Steps study also includes a process evaluation of the two Green Prescription interventions, an examination of barriers and motives for physical activity in the sample, and an evaluation of the perceptions of primary care physicians in relation to delivering physical activity counselling.

## Results

The results from this trial will be available late in 2009.

## Discussion

The study utilises an existing primary care physical activity program in New Zealand and compares it to the same program modified with the use of a pedometer as an instant feedback and motivational tool. If the pedometer-based Green Prescription increases physical activity and quality of life, and improves functional status, blood pressure and weight status in older adults, their utilization of health services is likely to decrease. Other benefits could include improved cardiovascular health and reduction of risk for a range of non-communicable lifestyles diseases.

## Competing interests

The authors declare that they have no competing interests.

## Authors' contributions

GSK and GMS conceived the study, contributed to study design and methodology, supervised the overall study conduct, directed analyses and assisted in manuscript preparation. NK assisted in study design, direction of analyses, and preparation of the manuscript. NG assisted in data management and analysis and preparation of the manuscript. PJS assisted in study design and preparation of the manuscript. TA assisted in the study design (economic analysis) and in preparation of the manuscript. AP participated in data collection and management of data. All authors read and approved the final manuscript.

## Pre-publication history

The pre-publication history for this paper can be accessed here:

http://www.biomedcentral.com/1471-2458/9/404/prepub
